# Publicações Científicas Relevantes e com Autonomia: O Caminho das Revisões Sistemáticas e Metanálises

**DOI:** 10.36660/abc.20240630

**Published:** 2024-11-21

**Authors:** Camila Mota Guida, Rhanderson Cardoso

**Affiliations:** 1 Instituto Dante Pazzanese de Cardiologia São Paulo SP Brasil Instituto Dante Pazzanese de Cardiologia, São Paulo, SP – Brasil; 2 Brigham and Women's Hospital Boston Massachusetts EUA Brigham and Women's Hospital, Boston, Massachusetts – EUA

**Keywords:** Publicações, Comunicação e Divulgação Científica, Estudantes de Medicina, Sucesso Acadêmico

De acordo com a *Association of American Medical Colleges* (AAMC), em 2023, o número médio de publicações por candidato aprovado para residência médica nos EUA foi de seis para medicina interna, oito para neurologia, nove para cirurgia geral e 19 para otorrinolaringologia.^1^ Igualmente, no Brasil, Reino Unido, Austrália e outros países, os processos seletivos para residência médica ou pós-graduação frequentemente consideram as publicações como um critério importante de avaliação.

Nos Estados Unidos, há uma cultura estabelecida de participação em projetos de pesquisa entre aspirantes à medicina, ainda antes de ingressarem na faculdade de medicina ( *medical school* ). Além disso, é comum que os estudantes norte-americanos de medicina aproveitem as férias de verão, entre o primeiro e o segundo ano, para trabalhar em laboratórios de pesquisa básica, translacional ou clínica, visando melhorar suas chances de serem admitidos em residências médicas de excelência.^2^

No Brasil, no entanto, profissionais em início de carreira encontram dificuldades significativas para a produção científica. Os recursos destinados à pesquisa são limitados e o excesso de burocracia representa um grande obstáculo. Relatos indicam que cerca de um terço do tempo dos pesquisadores brasileiros é consumido por problemas burocráticos, especialmente relacionados à compra de materiais e insumos.^3^ Esses desafios se refletem no fato de que, embora o Brasil seja o quinto país com maior número de universidades,^4^ o país ocupou em 2023 apenas a 23ª posição em número de publicações científicas na área da saúde e ciências naturais, ficando atrás de países como Alemanha, França e Coreia do Sul, que possuem menos universidades e populações menores.^5^ Além disso, a pesquisa no Brasil favorece uma hierarquia acadêmica que privilegia pesquisadores com dedicação exclusiva à ciência, como mestres, doutores e pós-doutores, o que torna ainda mais difícil para estudantes de medicina e médicos em início de carreira obterem publicações científicas relevantes.

Como, então, atender à crescente demanda de médicos e estudantes de medicina por aprendizado em metodologia científica e, principalmente, por resultados de pesquisa? O número cada vez maior de faculdades de medicina agrava um problema já existente até mesmo nas instituições de ensino e nos programas de residências mais tradicionais do país:^6^ a falta de infraestrutura, apoio institucional e *conhecimento* para condução de estudos científicos de qualidade. Corrigir esse problema exige, antes de tudo, uma mudança cultural, de valorização de pesquisa, construção curricular e apoio financeiro – tanto público quanto privado – voltados para a promoção de pesquisa. Parcerias entre *Academic Research Organizations* (ARO), em franca expansão no Brasil, e instituições de ensino poderiam servir como um canal para expor o estudante de medicina ou residente a oportunidades de pesquisa.

Enquanto tais mudanças estruturais não acontecem, quais as opções disponíveis atualmente para o estudante de medicina ou médico participar em pesquisa e publicações científicas? Em primeiro lugar, relatos de caso ou séries de caso. Esse é o caminho de entrada clássico para alguma apresentação em congresso ou publicação. Porém, há um problema com essa estratégia: o baixo valor acadêmico. Relatos de caso agregam pouco à literatura, na formação, e na competitividade de um currículo médico. A segunda opção é uma vaga de *research fellow* no exterior ou de pesquisa em grandes centros acadêmicos do Brasil. Essa estratégia é obviamente limitada em escala e fora do alcance da grande maioria de estudantes e médicos, pois há relativamente pouca disponibilidade para estas vagas.

Há uma terceira opção que preenche todos os pré-requisitos desejáveis para inserção do estudante de medicina brasileiro em pesquisa científica: valor acadêmico, disponibilidade e autonomia. Trata-se da revisão sistemática e metanálise. Quando conduzida adequadamente e incluindo estudos de alta qualidade, a metanálise oferece maior poder estatístico e melhor estimação da magnitude dos efeitos analisados. Menos dependente de fatores externos em comparação a outras modalidades de publicação, a elaboração de uma metanálise exige um conjunto particular de habilidades técnicas, como leitura crítica de evidências, interpretação de dados, metodologia padronizada de condução de revisões sistemáticas e análise estatística. Além de seu alto impacto potencial na literatura, o desenvolvimento e publicação de metanálises constituem uma forma de produção científica de baixo custo e sem as barreiras burocráticas frequentemente enfrentadas por pesquisadores em início de carreira.

Entre os exemplos, destaca-se o de *Bulhões et al.: Catheter ablation versus medical therapy for atrial fibrillation in patients with heart failure with preserved ejection fraction: A systematic review and meta-analysis.* ^7^ Essa publicação feita no Heart Rhythm compara ablação de fibrilação atrial com tratamento medicamentoso em pacientes com fibrilação atrial e insuficiência cardíaca de fração de ejeção preservada. O primeiro autor, Elisio Bulhões, é estudante de medicina do 4° ano em Redenção, Pará. Os coautores incluem estudantes de medicina na Universidade Estadual do Amazonas, da Universidade Federal de Ciências da Saúde de Porto Alegre, um residente no Dante Pazzanese e um cardiologista em Goiás.

Outro exemplo foi publicado por *Moreira et al.: Direct oral anticoagulants versus antiplatelet therapy following transcatheter aortic valve replacement in patients without prior or concurrent indication for anticoagulation: A meta-analysis of randomized studies.* ^8^ Essa publicação feita no Catheter Cardiovascular Interventions, é de Matheus Moreira, na época interno de medicina em Natal, Rio Grande do Norte. Os autores avaliaram o uso de anticoagulantes não antagonistas da vitamina K em pacientes pós-TAVI sem indicação concomitante de anticoagulação.

Essas publicações em revistas bem-conceituadas da eletrofisiologia e cardiologia intervencionista tiveram a participação e liderança de estudantes de medicina e médicos de diversos lugares do Brasil e da América Latina, sem experiência prévia em pesquisa. Isso foi possível porque eles aprenderam a conduzir revisões sistemáticas e metanálises, uma ferramenta poderosa que permite a publicação de artigos relevantes com autonomia.

Ter autonomia em pesquisa não significa fazer sozinho, claro. Esses trabalhos foram conduzidos em bons grupos, com colaboração entre todos os coautores. Porém, os autores tinham autonomia para conduzir cada uma das etapas da revisão sistemática e metanálise. A ideia de comparar ablação vs. tratamento medicamentoso em pacientes com FA e insuficiência cardíaca com fração de ejeção preservada foi do primeiro autor, Elisio Bulhões, estudante no interior do Pará. Além da própria ideia, ele tem domínio para fazer todas as demais etapas: especificar os critérios de elegibilidade, criar uma estratégia de busca efetiva, triagem dos estudos, extração de dados, estatística, análise de risco de vieses, e submissão para a revista. Ou seja, o trabalho foi feito com um grupo forte de coautores, que colaboraram nas diversas etapas, mas sem depender de nenhuma instituição ou de alguma pessoa específica para realizar qualquer etapa. As revisões sistemáticas e metanálises também não dependem de um comitê de ética para aprovação, termo de consentimento, coletas de dados de prontuários ou seguimento individual de pacientes.

É importante ressaltar que isso em nada diminui a importância de pesquisa primária, dos ensaios clínicos, coortes, estudos epidemiológicos etc. Os fatos apresentados demonstram apenas o enorme potencial da pesquisa secundária através de revisões sistemáticas e metanálises como uma porta de entrada acessível para pesquisa e publicações relevantes pelo estudante de medicina e médico brasileiro, independente de área geográfica, universidade, filiação acadêmica, infraestrutura, apoio local para pesquisa, ou qualquer outro fator limitante.

Os exemplos citados aqui são publicações feitas por estudantes, residentes, médicos generalistas ou especialistas, alunos do Meta-Analysis Academy. Esse programa de treinamento em revisões sistemáticas e metanálises foi criado pelo Dr. Rhanderson Cardoso, médico formado pela Universidade Federal de Goiás, cardiologista pelo Johns Hopkins Hospital e especialista em imagem cardíaca pelo Brigham and Women's Hospital, Harvard Medical School. Motivado pelo impacto que as metanálises tiveram em sua própria carreira, abrindo oportunidades excelentes de residência, *fellowship* , mestrado e docência nos EUA, ele criou o programa em 2022 para oferecer a mesma possibilidade para médicos e estudantes de medicina, inicialmente no Brasil e depois internacionalmente. Atualmente, o Meta-Analysis Academy possui alunos em 80 países, com mais de 1100 participações dos alunos em publicações científicas indexadas e 1900 presenças em abstracts apresentados em congressos nacionais ou internacionais.

Entre as conquistas na cardiologia, alunos do Meta-Analysis Academy já apresentaram mais de 100 abstracts nos congressos do American Heart Association, American College of Cardiology e European Society of Cardiology. As publicações incluem revistas como o JAMA Cardiology, Journal of the American College of Cardiology (JACC), Journal of Heart and Lung Transplantation, Circulation: Cardiovascular Imaging, International Journal of Cardiology, e American Journal of Cardiology, entre outros.

Vale ressaltar que as oportunidades em revisão sistemática e metanálise vão muito além da cardiologia. Lucas Pereira, aluno na Escola Bahiana de Medicina, foi primeiro autor na revista Anaesthesia and Analgesia, avaliando estratégias de reposição volêmica em transplantados renais.^9^ Rafael Morgado, estudante de medicina na Universidade Federal de Santa Catarina, foi primeiro autor em publicação no European Journal of Trauma and Emergency Surgery, comparando manejo cirúrgico vs. conservador em pacientes com tórax instável.^10^ Júlia Pontes, estudante de medicina no Rio de Janeiro, foi primeira autora na revista Neurosurgery, uma das melhores revistas sobre neurocirurgia, avaliando a eficácia e segurança de clazosentan pós-hemorragia subaracnóidea.^11^ Arthur Petrucci, no 2° ano de medicina em João Pessoa, Paraíba, publicou uma metanálise comparando cetamina vs. terapia eletroconvulsiva para episódio depressivo maior na revista Psychiatry Research.^12^ Eduardo Barbosa, interno em Anápolis, Goiás, foi primeiro autor no Gastrointestinal Endoscopy, revista de alto impacto na gastroenterologia, comparando drenagem biliar guiada por ultrassom endoscópico (EUS) ou colangiopancreatrografia retrógrada endoscópica (ERCP) em pacientes com obstrução tumoral.^13^

O impacto dos resultados em pesquisa na carreira desses alunos é imensurável. Além do conhecimento adquirido, muitos já conseguiram um salto na carreira valendo-se de uma aplicação robusta do ponto de vista de pesquisa. São dezenas que alcançaram residência ou *fellowship* nos EUA, inclusive em especialidades competitivas, como cardiologia, ginecologia e obstetrícia e cirurgia geral. Muitos conseguiram mestrado ou doutorado, inclusive em instituições internacionais, como University of British Columbia e Johns Hopkins University, até mesmo com bolsa integral por mérito em publicações. Outros seguiram pelo caminho de pesquisa através de um *research fellowship* em centros de excelência, como o Minneapolis Heart Institute, University of Michigan e Harvard Medical School.

Outro benefício intangível da pesquisa é a conexão que se forma entre um estudante capacitado e um médico especialista que atua como mentor. A habilidade de ter ideias e criar bons projetos para revisão sistemática e metanálise abre a possibilidade para o próprio aluno convidar colaboradores e mentores para esses projetos, sejam eles internos ou externos à própria instituição do aluno. Estudantes de medicina brasileiros, nos exemplos citados acima e em outros, convidaram médicos de centros de excelência nacionais e internacionais para serem seus colaboradores, como Universidade de São Paulo, A.C. Camargo Cancer Center, San Raffaele Hospital, Cleveland Clinic e Harvard Medical School, entre outros.

Concluindo, a revisão sistemática e metanálise é uma ferramenta poderosa para inserção do estudante de medicina e médico brasileiro em pesquisa de qualidade, com autonomia, seja nos grandes centros ou no interior do Brasil, em instituições públicas ou privadas. Essa metodologia de pesquisa e os resultados concretos dela, com apresentações em grandes congressos e publicações na literatura mundial, elevam o conhecimento do aluno, criam pontes para colaborações com grandes médicos e pesquisadores e, por fim, abrem grandes portas na carreira para residências competitivas, pós-graduação, docência e outros caminhos de excelência, seja no Brasil ou internacionalmente.
