# Supplementary material for: Publicações Científicas Relevantes e com Autonomia: O Caminho das Revisões Sistemáticas e Metanálises
Source: Arq Bras Cardiol. 2024 Nov 21;121(12):e20240630. [Article in Portuguese] doi: 10.36660/abc.20240630 (PMC11634298; doi:10.36660/abc.20240630)
Supplement: Translation

# Translation: Relevant and Independent Scientific Publications: The Path of Systematic Reviews and Meta-Analyses

**DOI:** 10.36660/abc.20240630i

**Authors:** Camila Mota Guida, Rhanderson Cardoso

**Affiliations:** 1 São Paulo SP Brazil Instituto Dante Pazzanese de Cardiologia, São Paulo, SP – Brazil; 2 Boston Massachusetts USA Brigham and Women's Hospital, Boston, Massachusetts – USA

**Keywords:** Publications, Scientific Communication and Diffusion, Medical Students, Academic Success

According to the Association of American Medical Colleges (AAMC), in 2023, the average number of publications per candidate accepted into medical residency programs in the United States was six for internal medicine, eight for neurology, nine for general surgery, and 19 for otolaryngology.^1^Similarly, in countries such as Brazil, the United Kingdom, and Australia, publications are an important criterion in medical residency or postgraduate program evaluations.

In the United States, there is an established culture of participation in research projects among aspiring physicians even before entering medical school. Additionally, North American medical students commonly use the summer break between their first and second years to work in basic, translational, or clinical research laboratories to strengthen their applications for competitive residencies.^2^

In Brazil, however, professionals face significant difficulties in scientific production at the beginning of their careers. Resources allocated to research are limited and excessive bureaucracy represents a major obstacle. Reports indicate that around one-third of Brazilian researchers’ time is spent handling administrative tasks, particularly those related to procuring materials and supplies.^3^These challenges are evident in Brazil's research output: although Brazil ranks as the fifth largest country by number of universities,^4^in 2023 it placed only 23^rd^in scientific publications in health and natural sciences, behind countries like Germany, France, and South Korea, which have fewer universities and smaller populations.^5^Additionally, Brazil's academic system favors researchers focused solely on science, such as those with master's, doctoral, or postdoctoral degrees, making it even harder for early-career medical students and physicians to achieve significant scientific publications.

How, then, can the increasing demand from physicians and medical students for training in scientific methodology and research outcomes be met? The growing number of medical schools intensifies an existing issue in even the most established institutions:^6^a lack of infrastructure, institutional support, and resources for high-quality scientific research. Addressing this issue requires a cultural shift that values research, curriculum development, and financial backing from both the public and private sectors to promote research initiatives. Partnerships between Academic Research Organizations (ARO), which are rapidly expanding in Brazil, and educational institutions could provide students and residents with access to research opportunities.

While waiting for these structural changes, what current opportunities exist for medical students and physicians to engage in research and publications? First, case reports or case series. This is the classic entry route for a presentation at a conference or publication. However, there is a problem with this strategy: low academic value. Case reports add little to the literature, to qualification, and to the competitiveness of a medical curriculum. The second option is a research fellowship abroad or a research position in a major academic center in Brazil. This approach is limited in scale and accessible to only a minority of students and physicians due to the scarcity of available positions.

There is a third option that meets all the Brazilian medical students’ demands to participate in scientific research, namely academic value, availability and autonomy. This is the systematic review and meta-analysis. When done correctly and based on quality studies, meta-analyses offer greater statistical power and provide more accurate estimates of effect sizes. Compared to other publications, meta-analyses require fewer external resources, though they do demand technical expertise, including skills in evidence-based analysis, data interpretation, systematic review methodology, and statistical analysis. Besides their high potential impact on literature, meta-analyses are a low-cost scientific production method, avoiding many bureaucratic barriers often faced by early-career researchers.

Among the examples, the work by *Bulhões et al* stands out *.: Catheter ablation versus medical therapy for atrial fibrillation in patients with heart failure with preserved ejection fraction: A systematic review and meta-analysis.* ^7^This Heart Rhythm publication compares atrial fibrillation ablation with drug treatment in patients with atrial fibrillation and heart failure with preserved ejection fraction. The first author, Elisio Bulhões, is a 4th year medical student in Redenção, Pará. The co-authors include medical students from the State University of Amazonas, the Federal University of Health Sciences of Porto Alegre, a resident at Dante Pazzanese and a cardiologist in Goiás.

Another example is the work by *Moreira et al.: Direct oral anticoagulants versus antiplatelet therapy following transcatheter aortic valve replacement in patients without prior or concurrent indication for anticoagulation:* A meta-analysis of randomized studies.^8^This is another publication by Matheus Moreira in Catheter Cardiovascular Interventions, who was a medical intern in Natal, Rio Grande do Norte. The authors assessed the use of non-vitamin K antagonist anticoagulants in post-TAVI patients without concomitant indication for anticoagulation.

These publications in well-regarded journals of electrophysiology and interventional cardiology included contributions and leadership from medical students and physicians across Brazil and Latin America, many without prior research experience. They achieved this by learning to conduct systematic reviews and meta-analyses, a powerful approach enabling independent publication of impactful articles.

Having autonomy in research does not mean conducting it by oneself, of course. These studies were conducted in good groups, with collaboration between all co-authors. However, the authors had the autonomy to conduct each of the stages of the systematic review and meta-analysis. The idea to compare ablation versus drug treatment in patients with AF and heart failure with preserved ejection fraction originated with first author Elisio Bulhões, a student from rural Pará. Beyond the initial idea, he also handled each step of the process: defining eligibility criteria, developing a search strategy, screening studies, extracting data, performing statistical analyses, assessing bias risk, and submitting to a journal. The project involved a strong team of co-authors who supported various stages yet relied on no single institution or individual to complete any specific step. Systematic reviews and meta-analyses also avoid the need for ethics committee approval, consent forms, data collection from patient records, or individual patient follow-up.

This does not diminish the importance of primary research, clinical trials, cohort studies, and epidemiological research. Rather, it highlights the significant potential of secondary research through systematic reviews and meta-analyses as a practical entry point for Brazilian medical students and physicians to produce relevant research and publications, regardless of their geographic location, university, institutional support, or other constraints.

The examples here include publications by students, residents, general practitioners, specialists, and students of the Meta-Analysis Academy. This training program in systematic reviews and meta-analyses was founded by Dr. Rhanderson Cardoso, a physician trained at the Federal University of Goiás, a cardiologist from Johns Hopkins Hospital, and a cardiac imaging specialist from Brigham and Women's Hospital, Harvard Medical School. Inspired by the career-advancing opportunities that meta-analyses provided him in residency, fellowship, and teaching roles in the U.S., he created the program in 2022 to offer similar opportunities to medical students and physicians, first in Brazil and now internationally. Currently, the Meta-Analysis Academy has students in 80 countries, with over 1,100 student contributions to indexed publications and 1,900 abstracts presented at national and international conferences.

In cardiology alone, students have presented more than 100 abstracts at the American Heart Association, American College of Cardiology, and European Society of Cardiology conferences. Publications include prestigious journals such as JAMA Cardiology, Journal of the American College of Cardiology (JACC), Journal of Heart and Lung Transplantation, Circulation: Cardiovascular Imaging, International Journal of Cardiology, and American Journal of Cardiology, among others.

It is worth noting that opportunities in systematic review and meta-analysis go far beyond cardiology. Lucas Pereira, a student at Escola Bahiana de Medicina, was the first author of a publication in Anaesthesia and Analgesia, which assessed volume replacement strategies in kidney transplant patients.^9^Rafael Morgado, a medical student at the Federal University of Santa Catarina, led a study in the European Journal of Trauma and Emergency Surgery, comparing surgical and conservative management for patients with flail chest.^10^Júlia Pontes, a medical student from Rio de Janeiro, was the first author of an article in Neurosurgery, a top journal in the field, evaluating the efficacy and safety of clazosentan following subarachnoid hemorrhage.^11^Arthur Petrucci, a second-year medical student in João Pessoa, Paraíba, published a meta-analysis in Psychiatry Research comparing ketamine to electroconvulsive therapy for major depressive episodes.^12^Eduardo Barbosa, an intern from Anápolis, Goiás, served as the first author on a study in Gastrointestinal Endoscopy, a high-impact gastroenterology journal, comparing endoscopic ultrasound (EUS)-guided biliary drainage with endoscopic retrograde cholangiopancreatography (ERCP) in patients with tumor obstruction.^13^

The impact of the research results on the careers of these students is immeasurable. In addition to the acquired knowledge, many have already achieved a career leap by taking advantage of a robust application from a research point of view. Dozens have achieved residency or fellowship in the USA, including competitive specialties, such as cardiology, obstetrics and gynecology, and general surgery. Many students have earned master's or doctorate degrees, including from prestigious international institutions like the University of British Columbia and Johns Hopkins University, often through merit-based full scholarships tied to their publication records. Others have pursued research opportunities via fellowships at esteemed centers such as the Minneapolis Heart Institute, the University of Michigan, and Harvard Medical School.

An important benefit of research is the relationship that develops between a dedicated student and a medical specialist who serves as their mentor. The ability to generate ideas and develop robust projects for systematic reviews and meta-analyses allows students to invite collaborators and mentors to join their initiatives, whether from within their own institution or beyond. Brazilian medical students, as seen in the aforementioned examples, have successfully invited physicians from national and international centers of excellence to collaborate with them, including from the University of São Paulo. A.C. Camargo Cancer Center, San Raffaele Hospital, Cleveland Clinic and Harvard Medical School, among others.

In conclusion, systematic reviews and meta-analyses serve as powerful tools for integrating Brazilian medical students and physicians into high-quality research, offering autonomy, whether in large urban centers or in rural areas of Brazil, in both public and private institutions. This research methodology, along with its tangible outcomes, such as presentations at major conferences and publications in global literature, enhances students’ knowledge, fosters collaborations with prominent physicians and researchers, and ultimately opens doors to competitive residencies, postgraduate studies, teaching opportunities, and other pathways to excellence, both in Brazil and internationally.
